# Exploring the Beam Squint Effects on Reflectarray Performance: A Comprehensive Analysis of the Specular and Scattered Reflection of the Unit Cell

**DOI:** 10.3390/s24051438

**Published:** 2024-02-23

**Authors:** Manzoor Elahi, Amir Altaf, Slawomir Koziel, Anna Pietrenko-Dabrowska

**Affiliations:** 1 Department of Engineering, Reykjavik University, 102 Reykjavik, Iceland; koziel@ru.is; 2 Millibeam, Melbourne, VIC 3053, Australia; amiraltafdgu@gmail.com; 3 Faculty of Electronics, Telecommunications and Informatics, Gdansk University of Technology, 80-233 Gdansk, Poland; anna.dabrowska@pg.edu.pl

**Keywords:** reflectarray, beam squint, specular reflection, scattered reflection

## Abstract

In this article, the phenomena of beam deviation in reflectarray is discussed. The radiation pattern of the unit cell, which plays a vital role in shaping the beam of the reflectarray, is analyzed by considering undesired specular and scattered reflections. These unwanted reflections adversely affect the pattern of the single unit cell, thereby reducing the overall performance of the reflectarray. To conduct our investigations, three cases of reflectarray—i.e., (i) a center-fed with broadside beam (Case-I), (ii) a center-fed with the beam at 30° (Case-II), and (iii) off-center-fed with the beam at 30° reciprocal to feed position with reference to the broadside direction (Case-III)—are simulated. Different degrees of beam deviation are analyzed in each reflectarray by assessing the radiation pattern of a single element. The simulation results shows that maximum of 0°, 3.4°, and 0.54° beam squint across the bandwidth found in Case-I, Case-II, and Case-III, respectively; this leads to aperture efficiencies of 31.2%, 11.9%, and 31.2%, respectively. The significance of specular reflections is further confirmed by half (left half and right half) aperture analysis of Case-II. This involves simulating the half-plane aperture illuminated by horn antenna, resulting in a distinct beam angle at the same frequency. However, deviations of −4.71 to +4.1 for the left half aperture and −1.82 to +1.1 for the right half aperture are noticed. Although the analysis specifically focuses on the three cases of the reflectarray, the proposed methodology is applicable to any type of reflectarray. The study presented in this work provides an important insight into the practical aspects of reflectarray operation, particularly in terms of quantifying undesirable effects that are normally overlooked in the design of this class of arrays. To achieve a good performance, a new design of the dielectric loaded horn feed is proposed. This design approach is both simple and applicable to any reflectarray, with the added benefit of maintaining a low profile for the RA. Moreover, this work holds significant potential for remote sensing satellite systems as beam deviation can adversely impact data collection accuracy and compromise observation precision, resulting in distorted images, reduced data quality, and overall hindrance to the system’s performance in capturing reliable information.

## 1. Introduction

Reflectarrays (RAs) are high-gain antennas which are essential parts of satellite communication systems due to their attractive features such as low profile, reduced cost, and the ease of manufacturing and deployment. However, RAs suffer from narrow bandwidth, which is predominantly due to the nonlinear reflection phase response of the elements. Considerable research has been carried out to solve this issue [1,2,3,4,5,6,7]. High-gain RAs are good candidates for modern satellite communication systems and future technologies due to their beam steering capability [8,9,10]. However, for RAs with narrow beam widths, beam squint versus frequency can be a serious problem. When a single feed is used to illuminate the aperture, a frequency-dependent beam squint appears as a deviation of the main beam from the desired direction, which is responsible for the gain loss in the desired direction, low-gain bandwidth (GBW), and the efficiency of the RA [11]. In the context of wireless sensor networks, the loss in gain and efficiency becomes particularly critical. Ambient noises present along the transmission path corrupt audio signals, leading to a low signal-to-noise ratio. This necessitates the implementation of signal enhancement techniques. Consequently, a significant amount of computational time and power consumption is required to perform noise reduction, especially when dealing with large amount of data transmission. Various approaches, as outlined in [12], have been employed to optimize power consumption and time complexity in addressing this challenge. Therefore, it is necessary for any antenna system to exhibit beam with the peak gain in the desired direction.

Over the past decades, very few articles have been published regarding the beam deviation in RA. In [13], a geometrical analysis has been used to illustrate that a different aperture phase distribution at frequencies other than the designed frequency results in the beam squint. Further, it is demonstrated that the beam deviation with respect to frequency can be reduced if the beam is oriented in the specular direction. A similar demonstration has been reported in [14]. Unlike the above studies, a full-wave receive mode analysis is conducted in [15], which shows that the phase center (focal point) of the RA changes with frequency. Another factor that affects the beam direction is the F/D ratio, where F is the feed position from the center of the RA aperture and D is the maximum aperture dimension. The effect of the F/D on the beam squint has been reported in [16]. A small F/D ratio increases the number of phase overlaps on the aperture, which increases the phase error. The impact of the unit cell size on the performance of the RA has been investigated in [17,18]. A small unit cell size reduces the phase errors on the aperture, which results in a high gain across the bandwidth. The specular reflection is an important phenomenon to be considered in beam tilting. Ref. [19] demonstrates that the effects of the specular scattered field in the off-specular RA is due to the errors in the phase shift. The phase error arises from the assumption of the infinite array of the same size of the unit cell (UC) during simulation. On the contrary, in practical RA realizations, the size of the unit cell varies progressively from larger to smaller and suddenly changes to the largest one. Beam deviation across the bandwidth can be suppressed by utilizing the two horns at the mirror image position with respect to the beam [20]. All of the above studies are based on the fixed beam direction. Recently, a study in [21] improved the scanning performance by adapting the hexagonal geometry of the unit cells. The aforementioned method attempted to reduce the beam squint in RA. However, these techniques are lacking a comprehensive examination of the root cause, which is the radiation characteristic of the single UC. It is noteworthy to mention that the beam squint is an intrinsic property of the RA and cannot be eliminated but can only be reduced.

In this paper, the beam squint in the RA is investigated in detail, based on the radiation pattern of the UC under the unwanted reflections, i.e., specular reflection, scattering effect. The unit cell considered here comprises a circular patch; however, the study is not limited to this particular geometry. First, the incident waves were impinged on the unit cell at various angles and the radiation patterns were analyzed at various frequencies in the given band (10.7–12.75 GHz). Next, the tilt in the main lobe of the UC pattern is illustrated by considering the effect of the specular reflection and scattering. Subsequently, the concept is applied to the entire RA that explains the squint in the main beam of RA. For better understanding, three RAs—center-fed with the main beam along the broadside direction (Case-I), center-fed with the main beam at 30° with respect to the broadside direction (Case-II), and RA with the feed at the mirror image of the main lobe at 30° (Case-III)—were designed and simulated using CST Microwave Studio. The basis of the three cases for the beam squint investigation is to summarize the various configurations in the literature that are either center-fed [22,23] or off-center-fed [3]. The comparison of the three cases is discussed in terms of the beam squint, gains, and the GBWs. The approach of this study identifies the root cause of the beam deviation from the desired direction in RA. Furthermore, Case-II is analyzed with half aperture of the RA to verify the specular reflection effect for various incident angles and the beam directions. The results obtained in this work can be considered a step towards a better understanding of undesirable effects (here, beam squint) affecting the operation of RAs, and ultimately leading to the development of methodologies for designing higher-performance reflectarrays.

## 2. Beam Squint in Reflectarrays

### 2.1. Design and Beam Scanning

**Figure 1:**
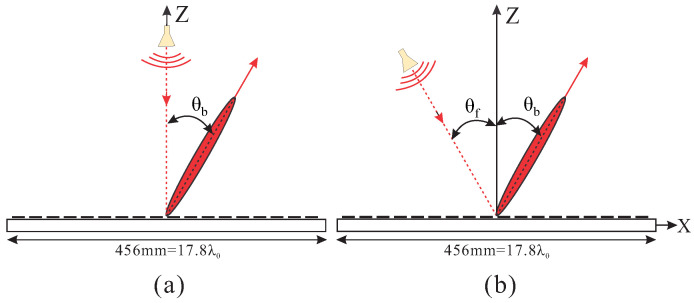
Schematic diagram of RA with beam oriented at 30°. (**a**) $\theta_{f}$° = 0°. (**b**) $\theta_{f}$° = 30°.

**Figure 2:**
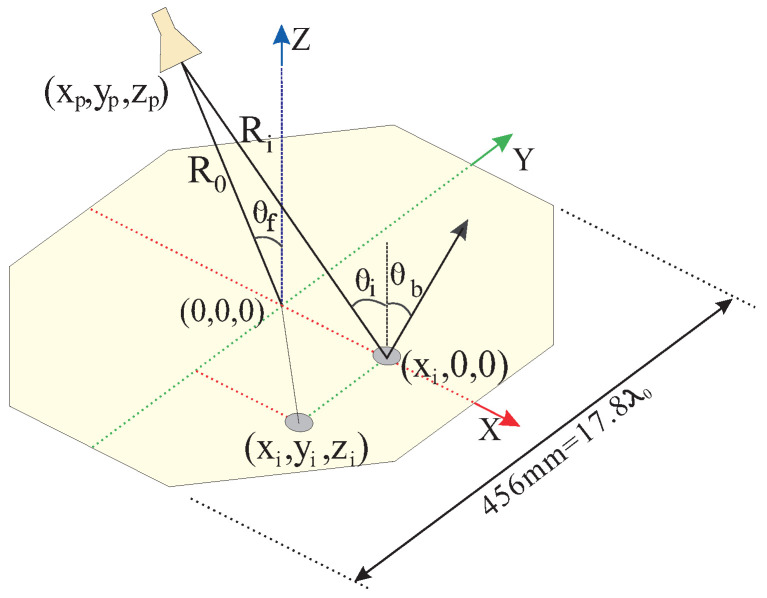
Schematic representation of RA and coordinate system.

Two RAs with center-fed and off-center-fed horns, as shown in Figure 1, are designed. The beams are aimed at 30° in both cases with the phase centers of the RAs located at ($X_{p}$, $Y_{p}$, $Z_{p}$) = (0, 0, 350) mm and (−175, 0, 303.108) mm, respectively. Mechanical rotation of the horn is adapted to control the beam steering. The aperture of the RA is an octagonal shape with a maximum dimension of 456 mm (17.8$\lambda_{0}$), where $\lambda_{0}$ is the wavelength in the free space at the center frequency 11.725 GHz. To avoid spillover losses, F/D = 0.765 is chosen to provide proper aperture illumination with an edge taper of about −10 dB. To design the RA, a schematic diagram is depicted in Figure 2. The realized phase reflection of each element on the aperture regarding the specific direction of the beam can be obtained by Equation [24]:

(1) $$\begin{array}{c} \phi_{i}=k_{0}\left(R_{i}-\left(x_{i}\cdot sin\left(\theta_{b}\right)\cdot cos\left(\phi_{b}\right)+y_{i}\cdot sin\left(\theta_{b}\right)\cdot sin\left(\phi_{b}\right)\right)\right)-k_{0}\cdot R_{0} \end{array}$$

where $k_{0}$ is the wave number in free space; $R_{i}$ is the spatial phase delay from the phase center of the array to the *i*th element. $x_{i}$ and $y_{i}$ are the coordinates of the *i*th element on the aperture. $\theta_{b}$ and $\phi_{b}$ represent the beam direction in space. $R_{0}$ is the distance between the phase center of the RA and the reference point (center of the RA aperture). In our case, $\phi_{b}$ = 0°; thus, (1) becomes

(2) $$\begin{array}{c} \phi_{i}=k_{0}\left(R_{i}-\left(x_{i}\cdot sin\left(\theta_{b}\right)\right)\right)-k_{0}\cdot R_{0} \end{array}$$

**Figure 3:**
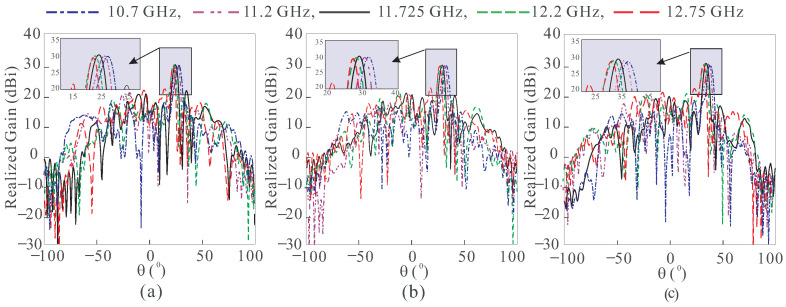
Simulated radiation patterns of the center-fed RA at (**a**) 25°. (**b**) 30°. (**c**) 35°.

**Figure 4:**
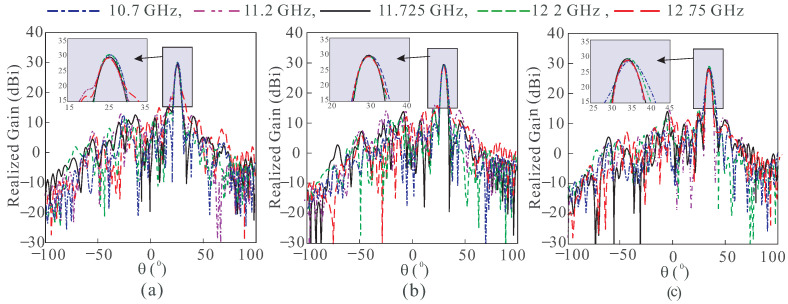
Simulated radiation patterns of the off-centered RA at (**a**) 25°. (**b**) 30°. (**c**) 35°.

To show the beam scanning performance in the desired bandwidth, the results of center-fed RA are shown in Figure 3a–c. The beam at the center frequency, i.e., 11.725 GHz, has negligible squint during scanning, however, it increases as frequencies become farther away from the center frequency. In the case of the off-centered RA, there is an insignificant beam squint at all frequencies, as shown in Figure 4a–c. Both the angles of the feed and the beam are mirror image to reduce the squint effect. Table 1 summarizes the beam squint for both RAs in Figure 1 for three scanning angles at five different frequencies. The squint for the center-fed RA is higher for all frequencies except at the center frequency, which results in the gain level below 30 dBi. In the off-center-fed RA, the beam squint is very small, not only at the center frequency but throughout the bandwidth. Thus, a negligible variation in the gain occurs due to which the gain level is maintained at around 30 dBi during the scanning.

### 2.2. Beam Squint Investigation

The beam squint minimization has been studied in [13]; here, it is based on the ray-tracing diagram of the wave. This analysis demonstrated that the $\theta_{f}$ = $\theta_{b}$ for the reduced beam squint. However, the following discussion provides more insight into the beam squint by considering the specular and scattering phenomena that affect the UC radiation pattern and give rise to the overall beam squint of the RA.

**Figure 5:**
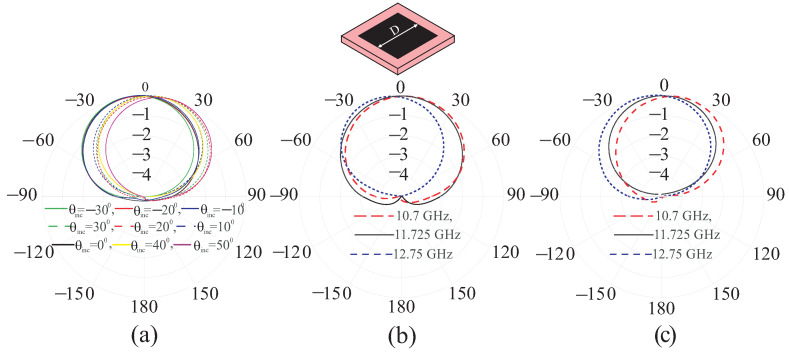
Normalized radiation patterns of the UC of size D = 4.632 mm at (**a**) various oblique incidence at 11.725 GHz; (**b**) normal incidence at 10.7 GHz, 11.725 GHz, and 12.75 GHz; (**c**) incidence at 30° at 10.7 GHz, 11.725 GHz, and 12.75 GHz.

Figure 5a predicted the normalized radiation patterns of the fix-sized UC at various oblique incidences, which shows that the main lobe of the pattern changes its direction. Similarly, the radiation patterns at normal incidence and oblique incidence at three frequencies are shown in Figure 5b,c, respectively, for a fixed size of the UC. The main lobe of the patterns at the off-centered frequencies has different directions, as the UC provides different phase reflections irrespective of the incident angles. This behavior of the unit can be theoretically explained as follows.

#### 2.2.1. Theoretical Model of the Unit Cell Reflection Characteristics

**Figure 6:**
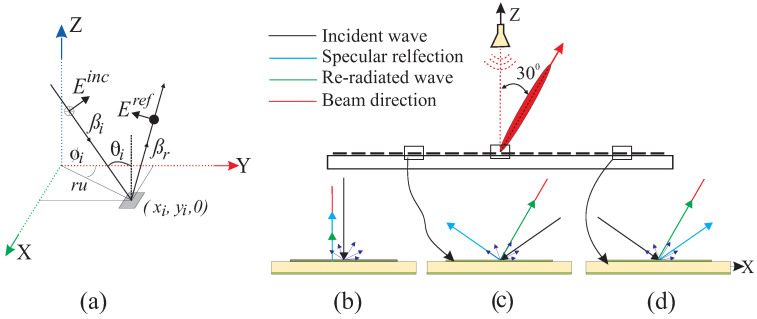
(**a**) Incident and reflected field of uniform plane wave at oblique angle on the unit cell; schematic diagram of the incident and reflected wave on the surface of the UC. (**b**) Normal incidence and reflection from the UC at the center of the center-fed RA with broadside beam. (**c**) Oblique incidence on the UC in LHA of the center-fed RA with beam oriented at 30°. (**d**) Oblique incidence on the UC in RHA of the center-fed RA with beam oriented at 30°.

As shown in Figure 6a, let $E^{inc}$ be the incident field illuminated on unit cell and $E^{ref}$ be the reflected field from the unit cell given by

(3) $$\begin{array}{c} E^{inc}=nE_{0}e^{-j\beta_{i}\cdot \left(x_{i}\cdot sin\theta_{i}\cdot cos\phi_{i}+y_{i}\cdot sin\theta_{i}\cdot sin\phi_{i}+z\cdot cos\theta_{i}\right)} \end{array}$$

(4) $$\begin{array}{c} E^{ref}=n\Gamma_{r}E_{0}e^{-j\beta_{r}\cdot \left(x_{i}\cdot sin\theta_{i}\cdot cos\phi_{i}+y_{i}\cdot sin\theta_{i}\cdot sin\phi_{i}+z\cdot cos\theta_{i}\right)} \end{array}$$

and

(5) $$\begin{array}{c} r_{u}=x_{i}\cdot sin\theta_{i}\cdot cos\phi_{i}+y_{i}\cdot sin\theta_{i}\cdot sin\phi_{i}+z\cdot cos\theta_{i} \end{array}$$

where n is the arbitrary direction of the incident and reflected *E*-fields, β=2π/λ is propagation constant at the design frequency $f_{r}$, and $\Gamma_{r}$ is the reflection coefficient of the unit cell. In (4), the reflection coefficient $\Gamma_{r}$ is the main factor that evaluates the behavior of the unit cell radiation pattern. For instance, in the lossless case of the unit cell, the reflected field is bounced back with the desired phase. Thus,

(6) $$\begin{array}{c} \Gamma_{r}=\Gamma_{0}e^{j\psi_{des}}=e^{j\psi_{des}} \end{array}$$

Therefore, the reflected *E*-field has the phase reflection factor $-\beta \cdot r+\psi_{des}$ with a zero phase error at the design frequency only, which can be written as

(7) $$\begin{array}{c} E^{des}=n\Gamma_{0}e^{j\psi_{des}}E_{0}e^{-j\beta_{r}\cdot r_{u}} \end{array}$$

However, at other frequencies in the band, there is a non-zero phase error as β·r results in a different phase values. This phase error leads to the variations in the E-field, impacting the main lobe of the unit cell. Now, consider the real scenario in which the reflection coefficient of the unit cell is a complex quantity, taking into account the dielectric losses, conductor losses, specular reflection, and unwanted scattered reflections, which implies that $|\Gamma_{r}|\neq 1$ and ψ is the sum of the phases of the specular and undesired scattered reflections. Each type of undesired reflection plays its role. To explain it, let an incident field $E^{inc}$ with phase $\phi_{i}$ from the feed impinges the UC. Some of the energy is re-radiated based on the UC dimension referred to as desired scattered field with reflection coefficient $\Gamma^{des}$, as discussed above. Some unwanted reflections additionally occur, such as a reflection from the bare grounded dielectric slab ($\Gamma^{spec}$) as well as scattered fields ($\Gamma^{scat}$). The reflection coefficient of the specularly reflected field depends on the oblique incidence, and properties of substrate and ground, whereas the reflection coefficient of the scattered field depends on the oblique incidence, shape, and non-resonant dimensions (length, width, diameter, etc.) of the UC. The undesirable reflection coefficient is given by [25]:

(8) $$\begin{array}{c} \Gamma^{undesired}=\Gamma^{spec}\left(\theta_{i},\phi_{i}\right)+\Gamma^{scat}\left(\theta_{i},\phi_{i},dimension\left(l_{i},w_{i}\right)\right) \end{array}$$

The *E*-field due to these reflection coefficients are given by

(9) $$\begin{array}{c} E^{spec}=n\Gamma_{0}e^{j\psi_{spec}}E_{0}e^{-j\beta_{r}\cdot r_{u}} \end{array}$$

(10) $$\begin{array}{c} E^{scat}=n\Gamma_{0}e^{j\psi_{scat}}E_{0}e^{-j\beta_{r}\cdot r_{u}} \end{array}$$

To characterize the scattering properties of the grounded dielectric slab loaded with conducting grating strips, it is common to use the co- and cross-polarization reflection coefficients with respect to the TE- and TM-polarizations. The TE- and TM-polarizations correspond to the ϕ and θ components of the field quantity, respectively. The $\Gamma^{spec}$ has θ and ϕ components, i.e., $\Gamma_{\theta \theta }^{spec}$, $\Gamma_{\phi \phi }^{spec}$; however, the co- and cross-polarization exists in case of the loaded dielectric slab [26]. Equations (9) and (10) can be written in terms of θ and ϕ for the TM- and TE-polarized reflected fields from the unit cell as:

(11) $$\begin{array}{c} E_{\theta }^{spec}=n\Gamma_{0\theta \theta }e^{j\psi_{spec}}E_{0\theta }e^{-j\beta_{r}\cdot r_{u}} \end{array}$$

(12) $$\begin{array}{c} E_{\phi }^{spec}=n\Gamma_{0\phi \phi }e^{j\psi_{spec}}E_{0\phi }e^{-j\beta_{r}\cdot r_{u}} \end{array}$$

(13) $$\begin{array}{c} E_{\theta }^{scat}=n\left[\Gamma_{0\theta \theta }E_{0\theta }+\Gamma_{0\theta \phi }E_{0\phi }\right]e^{j\psi_{scat}}e^{-j\beta_{r}\cdot r_{u}} \end{array}$$

(14) $$\begin{array}{c} E_{\phi }^{scat}=n\left[\Gamma_{0\phi \theta }E_{0\phi }+\Gamma_{0\phi \phi }E_{0\phi }\right]e^{j\psi_{scat}}e^{-j\beta_{r}\cdot r_{u}} \end{array}$$

The reflected field from the lossy unit cell is the sum of the field with all types of reflections. So, from (7), (9), and (10), the total reflected field $E_{t}^{ref}$ is

(15) $$\begin{array}{c} E_{t}^{ref}=E^{des}+E^{spec}+E^{scat} \end{array}$$

(16) $$\begin{array}{c} E_{t}^{ref}=n\left[e^{j\psi_{des}}+e^{j\psi_{spec}}+e^{j\psi_{scat}}\right]\Gamma_{0}E_{0}e^{-j\beta_{r}\cdot r_{u}} \end{array}$$

Thus, due to the inclusion of the phase of the undesired reflection coefficients, the reflected *E*-field leads to a different phase, resulting in a different direction of the main beam of the unit cell. That is the reason the main beam of RA deviates from the desired direction, and it is degraded at the off-centered frequencies.

Based on the above discussion, the reflection behavior of the UC is demonstrated in Figure 6, with the help of a ray tracing diagram. Figure 6b shows the UC at the center of the center-fed RA, producing a broadside beam. The specularly reflected fields align with the direction of the re-radiated field in this scenario. Figure 6c,d depict the UCs of the center-fed RA with the main beam at 30° in the left half aperture (LHA) and right half aperture (RHA), respectively. The re-radiated (desired scattering) waves are propagated in the beam direction for both the UCs. However, the effect of the specular reflection is more in the LHA as compared to the RHA. In LHA, the specular reflected fields carry energy in the direction by a greater angle than the re-radiated energy; however, in RHA, both the specular and re-radiated energies are propagated in directions by a small angle difference. Consequently, the directions of the reflected beams from both half planes will differ, as explained in the next section.

#### 2.2.2. Beam Squint in Different Cases of Reflectarray

**Figure 7:**
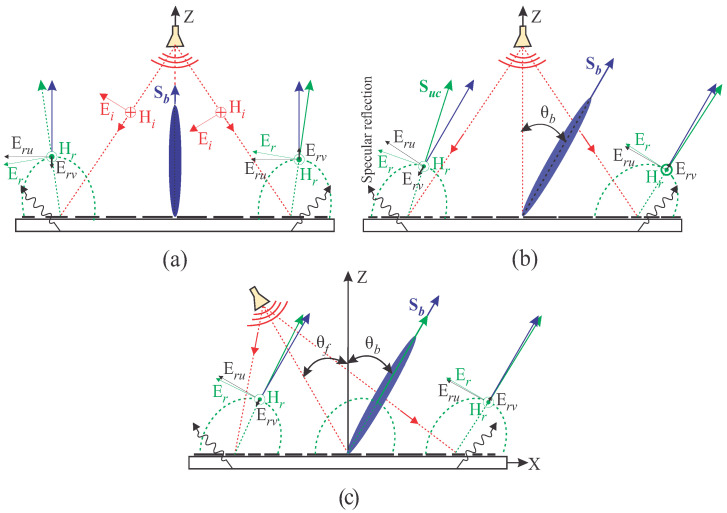
Schematic diagram of the RA along with the pattern of the UC: (**a**) center-fed RA with broadside beam; (**b**) center-fed RA with beam oriented at $\theta_{b}$ = 30°; (**c**) RA with feed oriented towards the mirror angle of the beam at $\theta_{b}$ = 30°.

The analysis in Figure 6 is applied to the three cases of RA. Figure 7 shows the schematic diagram of the RAs, along with the normalized radiation patterns of the UCs located at the mirror image distance relative to the center of the RA. $E_{i}$ and $H_{i}$ denote the incident electric and magnetic fields upon the surface of the UC, while $E_{r}$ and $H_{r}$ represent the net reflected fields from the surface of the UC. $S_{b}$ and $S_{uc}$ indicate the direction of the main lobe of the beam and the UC, respectively.

Figure 7a illustrates a center-fed broadside RA (Case-I). The reflected vectors $E_{r}$ and $H_{r}$ (green) specify the direction of $S_{uc}$ (green), which is the combination of the re-radiated energy from the UC and the specular reflection. Considering only the reflection phase of the UC at the center frequency, the re-radiated wave will have the same direction as $S_{b}$ (blue); however, unwanted scattering occurs at off-center frequencies. To study the effect of the unwanted reflections at the UCs in LHA and RHA, $E_{r}$ is analyzed with the beam direction, which has two rectangular components, $E_{ru}$ and $E_{rv}$ (black). The $E_{ru}$ contributes to the propagation of the wave in the broadside direction, whereas the $E_{rv}$ carries equal energy in opposite directions in the horizontal plane as the UCs have the same dimensions in both LHA and RHA. The same phenomenon occurs for the off-center frequencies without causing deviation of the beam. However, the degradation of the gain at the off-center frequencies is due to the phase error on the aperture.

A center-fed RA with the main beam at 30° off the broadside (Case-II) is given in Figure 7b. The UCs located at the mirror image distance from the center of the RA have different sizes, resulting in different amplitudes and phases of the main lobe ($S_{uc}$) of UCs at the same frequency. $S_{uc}$ in the LHA deviates more from $S_{b}$ compared to the UC in the RHA, as explained in Figure 6b,c. Intuitively, applying this concept to the whole array of elements, the final beam deviates from the exact beam direction. Specular reflection also affects the beam at the center frequency, but that is negligible. However, its effect is very significant at off-center frequencies. The effect of the specular reflection is minimized by the setup shown in Figure 7c (Case-III). In this case, $S_{uc}$ of the UCs in both halves of the aperture is not separated by a large degree from $S_{b}$, as observed in Case-II.

**Figure 8:**
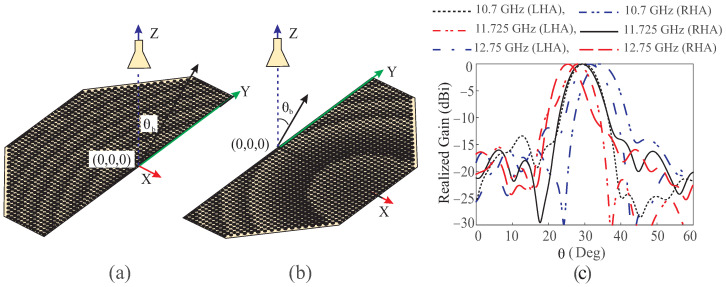
Simulation of Case-II RA: (**a**) with LHA; (**b**) with RHA; (**c**) normalized patterns of the main lobe for the half apertures RA at 10.7 GHz, 11.725 GHz, and 12.75 GHz.

To further explain the effect of the specular reflections in Figure 6, Case-II is simulated separately for the LHA and RHA, as depicted in Figure 8a,b, respectively. The beam direction at the same frequency differs for both half apertures, as illustrated in Figure 8c. Table 2 compares the beam directions at different frequencies in both half apertures. It is evident that the squint in the beam is larger for the LHA of the RA. Similarly, the squint will be larger for RHA if the beam is directed at $\theta_{b}$ = −30° in Case-II. Intuitively, if the RA in Figure 7a is simulated with half apertures, the same degree of beam squint will result for both the half apertures, and there will be no beam deviation in case of the beam from the full aperture. Similarly, the half apertures of RA in Figure 7c must have different squints but will differ at a minute level.

**Figure 9:**
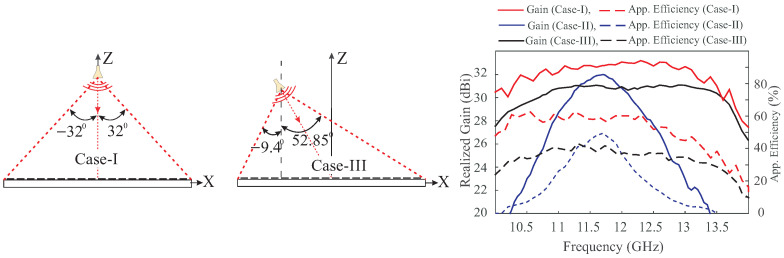
Farfield realized gain of Case-I, Case-II, and Case-III.

Summarizing the above discussion, Figure 9 compares the performance of the three cases in Figure 7 in terms of realized gain and aperture efficiencies. The aperture efficiencies are calculated by the following equation:

(17) $$\begin{array}{c} \eta_{app}=\frac{G\lambda^{2}}{4\pi A} \end{array}$$

where $\eta_{app}$, *G*, λ, and *A* are the aperture efficiency, realized gain, wavelength of the corresponding frequency, and area of the reflectarray, respectively. It can be observed that maximum aperture efficiencies of 62%, 50%, and 42% are obtained for Case-I, Case-II, and Case-III, respectively. Case-I exhibits the best gain performance, whereas Case-II shows good performance at center frequencies but performs poorly farther from the center frequency, resulting in a low 3 dB GBW of 11.9%. Case-III demonstrates intermediate gain performance but 3 dB GBW of 31.2%, similar to Case-I. The differences arise because the overall effect of specular reflection is canceled in Case-I due to the symmetry of the RA with broadside direction, whereas it is higher in Case-II.

In Case-III, the specular reflection is minimized, but the net specular reflection is not canceled. Similarly, the undesired scattering effect depends on the incident angle, shape, and non-resonant dimensions of the UC. By comparing Case-I and Case-III, the incident angle variations from the horn feed to the edges of the aperture are −32° to 32° and −9.4° to 52.85°, respectively. In Case-I, the incident waves are illuminated from the horn feed on both the LHA and RHA, the exact mirror image of each other, eliminating the overall effect of beam squint (as illustrated in Figure 7a). However, in Case-III, there is no half plane symmetry when illuminating waves from the horn feed. On the left of the horn feed, the incident waves make a maximum angle of −9.4°, while on the right of the feed horn, it is 52.85°. Thus, the squint in the beam due to unit cells on the left of the horn feed and the right of the horn feed is not balanced, which results in the overall tilted beam and degrades the performance of Case-III as compared to Case-I. Table 3 summarizes the main points of the above discussion.

## 3. Recommendations for Reducing the Beam Squint

The results presented in this work can be used to formulate design recommendations directed towards mitigation of the beam squint effects in reflectarrays. This can be realized by taking into account certain design parameters. Since RA is composed of unit cells illuminated by a feed horn, it is crucial to consider both the design of the unit cell and the design and position of the feed horn.

### 3.1. Unit Cell Design

#### 3.1.1. Unit Cell Periodicity

Squint caused by specular reflection primarily arises from the dielectric-loaded ground. Therefore, minimizing the exposure of the ground plane to feed illumination reduces specular reflection. Achieving this involves reducing the size of the unit cell, ensuring that the ground is less exposed to illumination from the horn antenna. However, decreasing the size while maintaining the full span of 360° makes the unit cell more phase-sensitive. Consequently, precision in fabrication technology becomes crucial for preparing the model, as even small changes in the unit cell’s dimensions can lead to an abrupt change in phase. This may result in phase errors adversely affecting the array performance in terms of controlling the beam direction.

**Figure 10:**
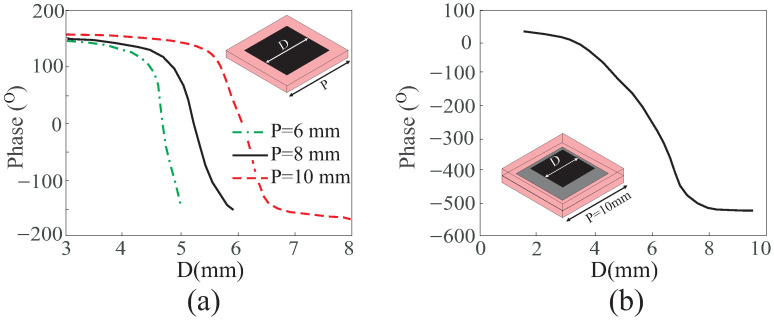
Phase range of unit cell: (**a**) single layer; (**b**) double layer.

Figure 10a presents simulation results for different periodicities of the UC, revealing that the phase range decreases and phase sensitivity (phase/unit length) increases with smaller periodicity. Therefore, a UC with low sensitivity and the smallest feasible size that is capable of fabrication is preferred. Additionally, achieving a large phase range and low sensitivity is possible through multilayered UCs. As depicted in Figure 10b, a phase range of more than 500° is obtained by a UC with two layers with slope not steeper than a single-layer UC. However, this is achieved at the expense of increased fabrication costs.

#### 3.1.2. Unit Cell Phase Range

Phase range covered by a unit cell is a crucial parameter that is especially significant in larger RAs. It is sufficient for a unit cell to cover a specific phase range on the RA’s aperture for a given F/D ratio. In the case of a large F/D ratio, the likelihood of phase overlaps on the aperture is low. However, for a small F/D ratio, the phase overlap increases, leading to higher phase errors. Extensive phase overlaps result in abrupt variations in unit cell dimensions, transitioning from smaller to larger sizes in each cycle. For RAs with small F/D ratios, it is advisable for the unit cell to encompass a larger phase range (with multiple phase overlaps) before making an abrupt transition from a small to a large size. A larger phase range prevents abrupt changes in unit cell dimensions, eliminating the occurrence of the phase errors. It is important to note that specular reflection is not susceptible to this type of phase error; thus, it is recommended to minimize phase errors.

**Figure 11:**
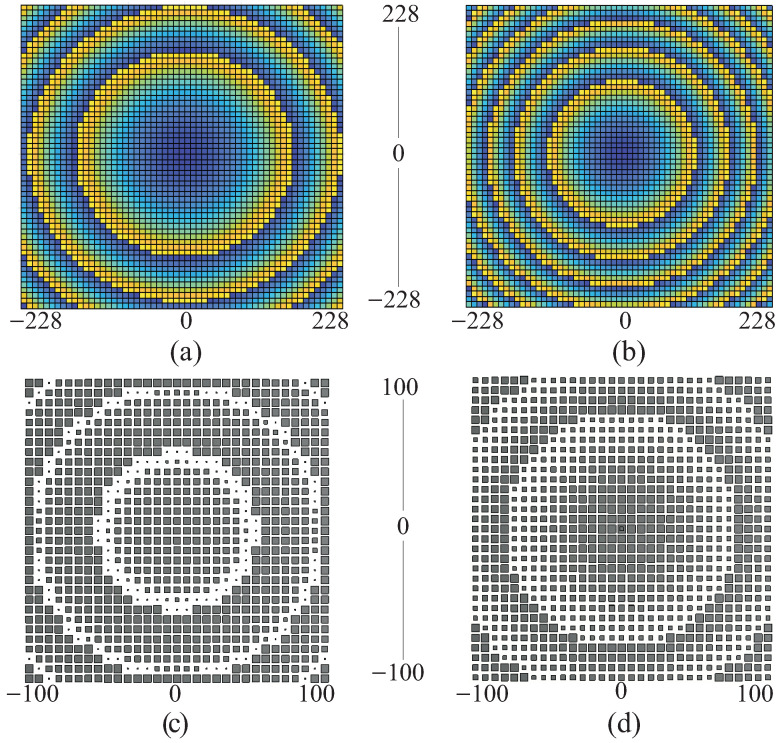
Phase distribution on the RA aperture with (**a**) F/D = 1, (**b**) F/D = 0.5, and patch distribution on the RA aperture with F/D = 0.5 for (**c**) UC with phase range of 360°, (**d**) UC with phase range of 500°.

Figure 11a,b depicts the phase overlaps on the RA’s aperture with F/D = 1 and F/D = 0.5, respectively. The RA with a larger F/D ratio shows lower phase overlaps. Therefore, RA with F/D = 1 has four phase overlaps and with F/D = 0.5 has six overlaps. Similarly, Figure 11c,d illustrates the unit cell pattern on the RA’s aperture with phase range of 360° and 500°, respectively. The variation in the sizes from smallest to the largest is minimal for the unit cell with larger phase range, i.e., two transition for phase range of 500° and three transition for a phase range of 360°.

In conclusion, to achieve optimal performance, it is recommended to use a smaller size of the unit cell with larger phase variations and lower phase sensitivity.

### 3.2. Design and Position of the Feeding Structure

The design and positioning of the feed are important parameters to reduce specular reflection in RAs. Initially, it is essential for the illuminated *E*-field intensity to exhibit a tapering of −10 dB at the edge of the RAs. The feed position is closely linked to the F/D ratio. By increasing the F/D ratio, the angle between the beam direction and the specularly reflected waves is reduced, aligning a major portion of the specularly reflected waves with the desired beam direction. This is more useful in cases where the beam is at the mirror image to the feed. However, a drawback of increasing the F/D ratio is that the profile of the antenna system becomes larger, making it impractical for deployment in various locations.

An alternative approach involves virtually increasing the F/D ratio, resulting in a much lower actual antenna profile. Two approaches can be adopted to achieve a larger F/D ratio—a Cassegrain RA and dielectric loaded horn feed RA.

#### 3.2.1. Cassegrain Reflectarray

**Figure 12:**
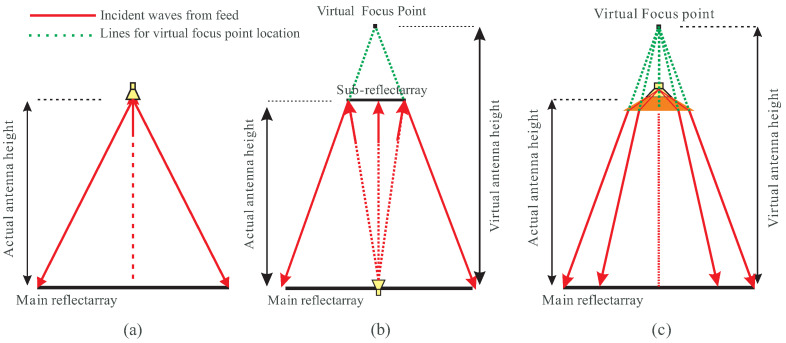
Schematic diagrams of (**a**) a simple horn-fed RA; (**b**) a Cassegrain RA; (**c**) a dielectric-loaded horn-fed RA.

The Cassegrain model presents an advantageous option for increasing the F/D ratio without enlarging the RA profile, as shown in Figure 12b. It is essential to note that the size and placement of the sub-reflectarray are carefully chosen to ensure no obstruction to the reflected waves from the main reflectarray. However, this Cassegrain structure introduces some challenges in terms of handling and adds complexity to the design. This complexity is particularly evident in shaping the pattern of the sub-reflectarray while considering the phase tapering of −10 dB and the F/D ratio on the main reflectarray aperture.

#### 3.2.2. Dielectric-Loaded Horn Antenna

**Figure 13:**
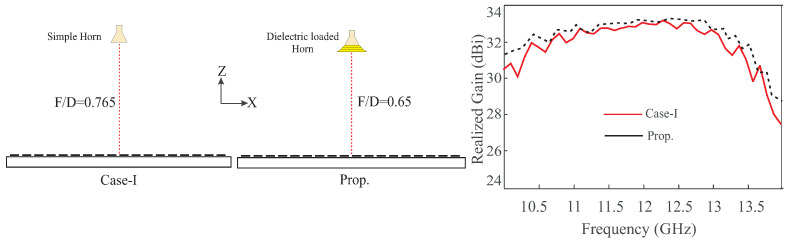
Geometry and comparison of the RA’s in Case-I and proposed design in terms of farfield realized gain.

This new design model is a relatively simple and easy to deploy and design. By taking into account the dielectric constant of the loaded dielectric structure, it becomes possible to control the wave in a manner that virtually increases the F/D ratio while keeping the system compact. In Figure 12c, the ray tracing diagram of the dielectric-loaded structure of the horn antenna is presented, demonstrating how the virtual F/D ratio can be enhanced. This structure, resembling a simple horn antenna is straightforward to deploy and design, making it easier to achieve a −10 dB tapering at the edges of the RA compared to the dual reflector structure found in the Cassegrain RA. To validate the proposed design, its performance is compared with the RA in Case-I in terms of farfield realized gain, as illustrated in Figure 13. Beams of both RAs are aimed in the broadside direction. It is evident that the overall gain is slightly improved by 0.2 dB to 0.5 dB across the band while maintaining a low profile with an F/D ratio of 0.65 as compared to the RA in Case-I. The reason for this improvement is the reduction in squint achieved by virtually increasing the F/D ratio while keeping the profile of the RA low. Another advantage of the proposed dielectric loaded horn is that enhancing F/D ratio along with −10 dB tapering at the aperture can easily be achieved by optimizing the loaded dielectric without affecting the parameters of the horn antenna. Therefore, a commercially available horn antenna can be used as a feeder for the RA.

Table 4 compares the merits and demerits of the different techniques used in RAs. The methods employed in [13,14,16,20] are based on the geometry of the RA. A commonly used approach in [13,14] aims to reduce beam squint by maintaining the beam in the same direction as the natural specular reflection. However, this geometry is not always desirable for all applications. Ref. [16] demonstrated that increasing the F/D ratio of RA reduces the beam squint and this method can be applied to various application but leads to a high-profile RA. In [20], a two-horn concept is introduced to reduce beam deviation. By using two horns at reciprocal angles, the effect of the specular reflection is canceled. However, this method is limited to the cases where the beam is desired in the broadside direction. Furthermore, using two horns makes the system heavy. Another technique presented in [17,18] is based on the design of the UC. A small size of the UC prevents most of the specular reflection that enhances the performance of the RA. However, in RA, a large phase range is desired to avoid the phase error on the aperture. Therefore, to achieve a large phase with small size of the UC, a multilayered structure is utilized that leads to the increased fabrication cost. On the contrary, if the small size of the UC is used with a single layer, sometimes it increases the phase sensitivity, requiring more precise fabrication technology to achieve the required phase. Considering the pros and cons, the recommended method has an edge over the previous techniques. By integrating the dielectric with the horn antenna, the RA has a low profile with virtually high F/D ratio that enhances the performance. This technique is very simple and can be utilized in every application.

## 4. Conclusions

Beam steering is one of the prominent applications in remote sensing satellite systems and future technologies. To ensure the high data rates without interference and with accuracy and precision in communication, there should be no deviation from the desired beam direction. This study investigated the beam squint in RA, based on the unit cell radiation pattern. First, the unit cell reflection behavior was studied by impinging the electromagnetic waves upon it at various incident angles. Then, different cases of the RA were simulated and various degrees of the beam squints were illustrated by focusing the radiation patterns of the single unit cell. Next, for further explanation of the specular reflection, Case-II (center-fed RA with the beam at 30°) was simulated with separate LHA and RHA, which shows different degrees of beam squint. Finally, the performance of the three cases has been compared in terms of the gains and GBWs. This work provides an important insight into practical aspects of reflectarray operation, especially in terms of quantifying undesirable effect normally neglected in the design of this type of arrays. The results obtained in this work can be regarded as a step towards a better understanding of undesirable effects, specifically the beam squint, which affect the operation of RAs. Addressing these concerns, several recommendations have been provided to enhance the performance of RAs, focusing on the design aspects of unit cells and feeds. These recommendations ultimately contribute to the development of methodologies for designing higher-performance RAs.

## Figures and Tables

**Table 1:** Comparison of the beam squint at different scanning angles for RA in Figure 1a,b.

	Type of RA	$\theta_{f}$°/Freq. (GHz)	10.7	11.2	11.725	12.2	12.75
Squint	Center-Fed	25	2.6	1.3	0	1.5	3
		30	2.8	1.5	0.1	1.2	2.9
		35	3.1	1.7	0	1.6	3.4
	Off-Center-Fed	25	0.4	0.4	0.1	0.1	0
		30	0.5	0.2	0	0	0.1
		35	0.4	0.2	0	0.2	0.54

**Table 2:** Comparison of the beam directions for the LHA and RHA in Case-II.

Frequency (GHz)	10.7	11.725	12.75
$\theta_{f}$°	0	0	0
$\theta_{b}$° (LHA)	34.1	29.2	25.29
$\theta_{b}$° (RHA)	31.1	29.5	28.18

**Table 3:** Performance comparison of the three cases of RA.

Case	Gain (dB)	3 dB GBW (%)	$\eta_{\mathrm{app}}$ (%)	Symmetry	$r_{\mathrm{net}}^{\mathrm{spec}}$	$r_{\mathrm{net}}^{\mathrm{scatt}}$
Case-I	33	31.2	62	*x*-, *y*-axis	0	0
Case-II	31	11.9	50	*x*-axis	High	High
Case-III	31.89	31.2	42	*x*-axis	Low	High

**Table 4:** Techniques used for reducing the beam squint in RA.

Ref.	Technique	Merits/Demerits
[13]	Beam aligned with specular reflection	Not desired everywhere
[14]	Beam aligned with specular reflection	Not desired everywhere
[16]	High F/D ratio	Bulky size
[17]	Reduced UC size	Multilayered structure used to cover 360° phase
[18]	Reduced UC size	Multilayered structure used to cover 360° phase
[20]	Two horns at reciprocal angles	Complex and heavy
[Prop.]	Virtually high F/D ratio	Reduced profile, applicable to any RA, and simple

## Data Availability

Data are contained within the article.
